# 

**Published:** 1891-08-15

**Authors:** 


					HOMAS'S'JJoSPr
)rwich hospital
?La sgqw Wes term Infirmabx
with EXTRA NURSING SUPPLEMENT.
No. 255. Vol. X.]
Saturday,
August 15 th, 1891.
[One Penny.

				

